# A Hybrid Imaging Platform(CT/PET/FMI) for Evaluating Tumor Necrosis and Apoptosis in Real-Time

**DOI:** 10.3389/fonc.2022.772392

**Published:** 2022-06-22

**Authors:** Yulin Kang, Xiaohui Zhai, Sifen Lu, Ivan Vuletic, Lin Wang, Kun Zhou, Zhiqiang Peng, Qiushi Ren, Zhaoheng Xie

**Affiliations:** ^1^ Institute of Environmental Information, Chinese Research Academy of Environmental Sciences, Beijing, China; ^2^ Department of Biomedical Engineering, College of Engineering, Peking University, Beijing, China; ^3^ Precision Medicine Key Laboratory of Sichuan Province and Precision Medicine Center, West China Hospital, Sichuan University, Chengdu, China; ^4^ State Key Laboratory of Proteomics, National Centre for Protein Sciences, Beijing Institute of Lifeomics, Bejing, China

**Keywords:** multi-modality system, animal imaging, *in vivo*-therapeutic approach, cancer, gene therapy

## Abstract

Multimodality imaging is an advanced imaging tool for monitoring tumor behavior and therapy *in vivo*. In this study, we have developed a novel hybrid tri-modality system that includes two molecular imaging methods: positron emission computed tomography (PET) and fluorescence molecular imaging (FMI) and the anatomic imaging modality X-ray computed tomography (CT). The following paper describes the system development. Also, its imaging performance was tested *in vitro* (phantom) and *in vivo*, in Balb/c nude mice bearing a head and neck tumor xenograft treated with novel gene therapy [a new approach to the delivery of recombinant bacterial gene (IL-24-expressing strain)]. Using the tri-modality imaging system, we simultaneously monitored the therapeutic effect, including the apoptotic and necrotic induction within the tumor *in vivo*. The apoptotic induction was examined in real-time using an ^18^F-ML-10 tracer; the cell death was detected using ICG. A CT was used to evaluate the anatomical situation. An increased tumor inhibition (including tumor growth and tumor cell apoptosis) was observed in the treatment group compared to the control groups, which further confirmed the therapeutic effect of a new IL-24-expressing strain gene therapy on the tumor *in vivo*. By being able to offer concurrent morphological and functional information, our system is able to characterize malignant tissues more accurately. Therefore, this new tri-modality system (PET/CT/FMI) is an effective imaging tool for simultaneously investigating and monitoring tumor progression and therapy outcomes *in vivo*.

## Introduction

Cancer is still one of the major causes of death worldwide. Global cancer rates are expected to reach 21.6 million new cases per year by 2030 (1). Recent developments in non-invasive imaging methods have significantly promoted tumor diagnosis and therapy in both clinical and preclinical settings. For example, using PET, SPECT, MRI, CT, optical devices, and ultrasound approaches, researchers are now able to simultaneously obtain morphological and functional characteristics of tumor tissues in patients or live animals (2). In addition, great progress has been made in understanding the mechanisms of tumor apoptosis (3, 4), one of the hallmarks of cancer progression. Using molecular imaging, scientists are now abale to monitor specific biochemical changes inside the cells in real-time (5, 6).

Most imaging-based analyses rely on radiolabeled tracers/probes to investigate the roles of apoptosis in cancer. In the early 2000s, several *in vivo* studies reported using ^99m^Tc-, ^125^I-, ^18^F-labelled annexin V for investigating cell apoptosis *in vivo* (7–9). Annexins are calcium-dependent phospholipid-binding proteins that bind to phosphatidylserine, targeting apoptotic cells. More recently, a new PET tracer (^18^F-ML-10) has been developed, which was specifically designed to detect apoptotic tissues *in vivo* (10). ^18^F-ML-10 has been designed to bind to and accumulate inside apoptotic cells and can be used to characterize the so-called apoptotic membrane imprint, thus offering an attractive molecular imaging approach in both preclinical and clinical work (11, 12). In addition, one of the most important advantages of using positron emission tomography (PET) over other imaging methods is that it allows data quantification.

Optical imaging offers a useful, safe, and low-cost, non-invasive method of imaging tumor cell death *in vitro* and *in vivo*. Luminescence imaging, operating on a wavelength scale above 600 nm (far-red and near infra-red luminescence), has proved to be a very useful technique for whole-body imaging in small animals such as mice (13). Currently, mKate2 and mNeptune are considered the most common far-red fluorescence luminescence proteins. The corresponding reporter genes can be stably integrated into the cellular genome and are characterized by a high quantum yield and stability (14, 15). In addition to luminescent tracers, fluorescent dyes such as cyanines (Cy7 and Cy7.5) as well as indocyanine green (ICG) are the most common dyes applied in non-invasive real-time whole-body imaging. These probes/dyes can be used for labeling reactions. For example, Cy-annexin with its emission peak above 750 nm has been proved useful for investigating cell apoptosis *in vivo* (16). Although Cy7 and Cy7.5 do have higher quantum yields, which is important when low imaging concentrations of target molecules, ICG has already been tested and used in a clinical setting (17). Furthermore, a recent discovery showed that ICG could achieve a high avidity to necrotic tissues owing to its affinity to lipoproteins and phospholipids (5). However, developing novel multi-modality techniques for *in vivo* detection of tumor functional characteristics, such as apoptosis, still presents unresolved challenges.

In this study, a custom-built tri-modality imaging system (PET/CT/FMI) was developed. Using the system, we simultaneously monitored the therapeutic effect of a novel gene therapy (a delivery of recombinant bacterial gene (IL-24-expressing strain), including the apoptotic and necrotic induction within the tumor *in vivo*. The apoptotic induction was examined in real-time using an ^18^F-ML-10 tracer; the cell death was detected using ICG, while a CT was used to evaluate the anatomical situation. In our previous study, we used a *Bifidobacterium breve *as a delivery vector of IL-24 gene therapy (B. breve-IL24) for head and neck squamous cell carcinoma *in vivo* and discovered that B. breve-IL24 offers a novel, safe, and clinically acceptable therapeutic approach for tumor therapy (18). Beneficial bacteria such as *Bifidobacterium strains* offer a straightforward, safe, and clinically relevant approach to delivering therapeutic proteins (19–21) locally to the hypoxic region of a tumor, which still resides externally with regard to the tumor cells themselves. Our self-developed tri-modality system has been demonstrated to be an effective imaging tool to simultaneously investigate and monitor tumor progression and therapy success *in vivo*, and might be useful for preoperative prediction and intraoperative monitoring of tumors in real-time.

## Materials and Methods

### System Design

**Figure1:**
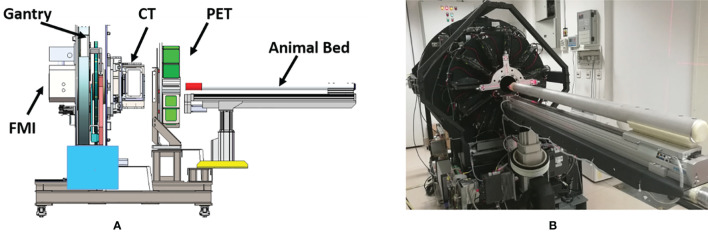
**(A)** Schematic representation of the tri-modality imaging system. **(B)** The actual appearance of the tri-modality imaging system.

The tri-modality imaging system has a modular design. The PET, CT, and FMI modules were initially developed separately and then integrated into the same gantry. The system includes five major components: gantry, CT, PET detector ring, FMI module, and the animal bed (**Figure 1**).

The CT scanning geometry uses a rotated source-detector pair when imaging living animals. It consists of a flat-panel CMOS detector (1512, DEXELA Inc., USA) and an X-ray tube (SB-80-250, Source-ray Inc., USA) with a 33 μm focal spot. The X-ray tube and the CMOS detector are mounted on a high precision translation stage which is mounted on the rotatable gantry. The PET module is made up of 22 detector modules arranged in a ring-like geometry with an interior diameter of 100 mm and an axial FOV of 32 mm. The FMI module included two common fluorescence imaging modes: fluorescence molecular tomography (FMT, 3D whole-body imaging of mice) and fluorescence reflectance imaging (FRI, 2D imaging of subcutaneous tissues). Both imaging modes rely on a tungsten-halogen lamp (71LT250/71PT250A, Beijing 7-Star, China) as an illumination source, while an sCMOS (ORCA-Flash 4.0, Hamamatsu, Japan) is used as the detection device. The whole setup is mounted on a surface plate which is also connected to the gantry module.

FMI and CT imaging modules share one rotation gantry. In addition, all three modalities (PET/CT/FMI) images were scanned on the same animal bed. The animal bed was mounted on a high precision motor-controlled translation stage which carries the imaging objects along with the different imaging modalities. Multi-modal image fusion was precisely done by co-registered positional data overlay. An anesthetics vaporizer and thermostatic equipment were reserved during the experiment for animal safety reasons.

### Phantom Tests

For system testing and calibration purposes, a phantom was constructed to evaluate all three imaging modalities. The solid, cylindrical phantom with an outer diameter of 30 mm and a length of 70 mm (made of polyformaldehyde) contained two 2 mm diameter holes of 35 mm depth. The center-to-center distance between the two holes was 10 mm. The phantom had an absorption coefficient of µa = 0.02 cm^-1^ and a reduced scattering coefficient of µs = 10.0 cm^-1^, which perfectly mimics tissue properties. The phantom images were used to calibrate the co-registration of the images obtained from the tri-modality system. For this, prior to the experiment, two capillary tubes were separately filled with indocyanine green (ICG, Merck Sigma-Aldrich, 10μL with 10 µM) and fluorodeoxyglucose (FDG, 50µL with 70µCi), were inserted into the holes of the phantom. The reconstructed CT, PET, and fluorescence images were used for registration.

PET images were first acquired over a time period of approximately 5 min using energy and coincidence windows of 350 – 650 keV and 12 ns, respectively. Subsequently, the phantom was moved to the field of view of the CT module. A CT acquisition was performed by employing the following parameters: X-ray voltage: 80 kVp, anode current: 450 μA, and exposure time of each of the 360 rotational steps was 50 ms. Finally, the fluorescence images were collected from 36 projections evenly along with the 360° rotation, and the integration time of the sCMOS camera was 4 s at each projection angle.

PET data were reconstructed after attenuation correction by using ordered-subsets expectation maximization (OSEM). A CT data were reconstructed by using a filtered back-projection algorithm. FMT data were reconstructed by the method of L1 norm regularization with modified FOCUSS (L1-FOCUSS). The obtained PET and FMT images were registered with the CT images, respectively.

### Synthesis of ^18^F-ML-10

ML-10 (2-(5-fluoro-pentyl)-2-methyl-malonic acid, MW=206 Da) is the prototypical member of a novel family of small-molecule detectors of apoptosis. The fabrication process of ^18^F-ML-10 was performed according to a previously described approach (22, 23).

^18^F-ML-10 was synthesized from its respective precursor, ML-10-mesylate, by a nucleophilic substitution reaction. For radiolabeling, ^18^F-fluoride delivered from a cyclotron was trapped on a quaternary ammonium cartridge, and excess 18H_2_O was removed. 18F was eluted with Kryptofix222 (Sigma-Aldrich) and azeotropic drying at 90°C under argon with acetonitrile resulting in dry ^18^F-fluoride. The precursor ML-10 mesylate in anhydrous acetonitrile was added for a 15-min reaction at 90°C. After dilution with acetonitrile, a silica Sep-Pak (Waters) was used to separate t-butyl–protected ^18^F-ML-10. Hydrolysis of the protecting groups was accomplished by treatment with trifluoroacetic acid (TFA):H_2_O (9:1) at room temperature for 15 min. Dilution with deionized water followed by semipreparative reversed-phase high-performance liquid chromatography (HPLC) (mobile phase of water:acetonitrile: TFA, 80:20:1 [v/v/v]; octyldecyl silane column [Phenomenex]; flow rate of 10 mL/min) provided purified ^18^F-ML-10. The product eluted at a retention time of 15 min. Organic solvents were removed after dilution with deionized water, and ^18^F-ML-10 was trapped on a Sep-Pak C18 column (Waters) under elution with absolute ethanol. The HPLC product fraction containing ^18^F-ML-10 was mixed with deionized water containing 1% acetic acid (100 mL), and the mixture was passed over a Sep-Pak C18 column. Flushing with absolute ethanol (1 mL) yielded the final product. The radiochemical yield of ^18^F-ML-10 was 40%255% at the end of synthesis (EOS), with a collected product yield of 30%240% after purification steps (EOS). The radiochemical purity was greater than 99%, and the specific activity was greater than 40.7 GBq/mmol (EOS). The overall time required for radiolabeling was about 75 min.

11.1 MBq (215 µL with 0.3 mCi) of ^18^F-ML-10 was injected through the tail vein to investigate the tumor apoptotic area. The specific activity of ^18^F-ML-10 is 51.6 MBq/g.

### Animal Experiment

Thirty specific pathogens free (SPF) grade BALB/c-nu/nu nude mice (6-8 weeks, weighing 18-22 g, male) were housed in an environment with a temperature of 22 ± 1 °C, relative humidity of 50 ± 1%, and a light/dark cycle of 12/12 hr, and were given water and food *ad libitum*. All animal studies (including the mice euthanasia procedure) were done in compliance with the regulations and guidelines of Peking University institutional animal care and conducted according to the AAALAC and the IACUC guidelines. Body weight was monitored and recorded weekly.

### Animal Imaging

**Figure 2:**
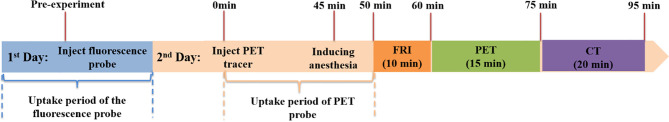
Timeline of animal experiments. Thirty SPF grade BALB/c-nu/nu nude mice (6-8 weeks, weighing 18-22 g, male) were used for imaging and WB. Among them, half of them (15) were assigned to the control group and 15 to the experimental group.

Thirty mice were used for imaging and Western Blot (WB). Among them, half of them (15) was assigned to the control group and 15 to the experimental group. The procedure of animal imaging consisted of the following steps: probe injection, probe uptake time, anesthesia procedure, and multi-modality image collection, as shown in **Figure 2**. The imaging process has been optimized in order to minimize the total experiment time.

In this study, the tri-modality imaging system was used to investigate the therapeutic effect (apoptotic and necrotic tumor induction) of a new recombinant bacterial strain on mouse head and neck xenografts *in vivo*. Briefly, two imaging probes were used for this purpose: firstly, in order to be able to investigate the necrotic tumor area, an ICG solution (8.0 mg/kg) was injected into the tail vein 24 h prior to the experiment. 8 mg/kg is a dose that takes into account both animal safety and fluorescence signal-to-noise ratio. Secondly, 11.1 MBq (0.3 mCi) of ^18^F-ML-10 was injected into the tail vein to detect the tumor apoptotic area. The timeline for the multi-modality imaging experiment was as follows (**Figure 2**): mice were abdominally injected with 2.5% avertin anesthetic (0.1ml/5g) and placed on the animal bed in a dorsal position. After 50 min, the fluorescence images were acquired with an exposure time of 1 s using appropriate filters (ex. filter with 769 nm, em. filter with 832 nm cut-off, *Semrock*). The raw data were acquired under the control of a baseline clamp and processed with the help of Andor SOLIS software. Immediately after fluorescence image recording, whole-body PET/CT images were acquired.

### Bacterial Tumor Therapy *in vivo*

The recombinant Escherichia coli–Bifidobacterium shuttle vectors pLW5 and pLW9, which include IL24 (835 bp) and green fluorescent protein (GFP, 930bp) fragments, respectively, were used to obtain B. breve-IL24 (pLW5-breve) and B. breve-GFP (pLW9- breve) transformant. B.breve-IL-24 transformants were obtained through electrotransformation. Transformed strains expressing IL-24 were analyzed by RT-PCR before starting the experiment, *as* described previously (18). Briefly, he WSU-HN6 cell line was used as reagents, which was authenticated by short tandem repeat profiling (STR) and free of mycoplasma contamination. (**Supplementary Figure S3**). 1x10^6^ WSU-HN6 cells were then subcutaneously injected into the mouse back (in 30 mice). When the tumor tissues reached 2-3 mm in diameter, mice were randomly assigned to a treatment or control group (10 mice in each group). The treatment group received 200 µL solution containing 1x10^8^ B.breve-IL-24 bacteria for the treatment group, while a 200 µL saline solution was given to the control group; injections were given by the tail vein twice per week for a total of 2 weeks. Tumor size was measured twice a week post-injection by using a standard caliper tumor volume calculation method: V_tumor_= (l x w²)/2. After treatments, all mice were imaged according to the *in vivo* imaging protocol mentioned above (see section” Animal imaging”). Then, the mice were euthanized, and tumor tissues were dissected and analyzed by Western blot and a histopathology assay. Detailed materials and methods for Western blotting and the histopathology assay are described in the “Electronic Supplementary Material” section. Cell necrosis and apopsos could be observed by HE staining. To be specific, volume expansion and deformation of groups of cells or some tissues can be observed. Cell membrane was observed damaged. Chromatin does not aggregate and appears flocculent. Organelles are enlarged or broken. The nuclei of apoptotic cells were pyknotic and fragmented, showing blue-black color, and their cytoplasm was light red. Normal cell nuclei are uniformly blue. While necrotic nuclei appear very pale blue or disappear.

### Statistical Analysis

Tumor size and real-time PCR results were expressed as mean ± standard deviation (SD). All calculations and analyses were performed using SPSS 19.0 software (SPSS Inc., Chicago, IL, USA). A P value <0.05 was considered as being statistically significantly different. All continuous variables were first tested for normality, and then the mean difference was tested according to the group t-test. The rank-sum test was performed when continuous variables did not pass the normality test. Categorical variables were tested by using the chi-square test. A p value <0.05 was considered to be statistically significant for all statistical methods.

## Results

### Phantom Experiments

**Figure 3:**
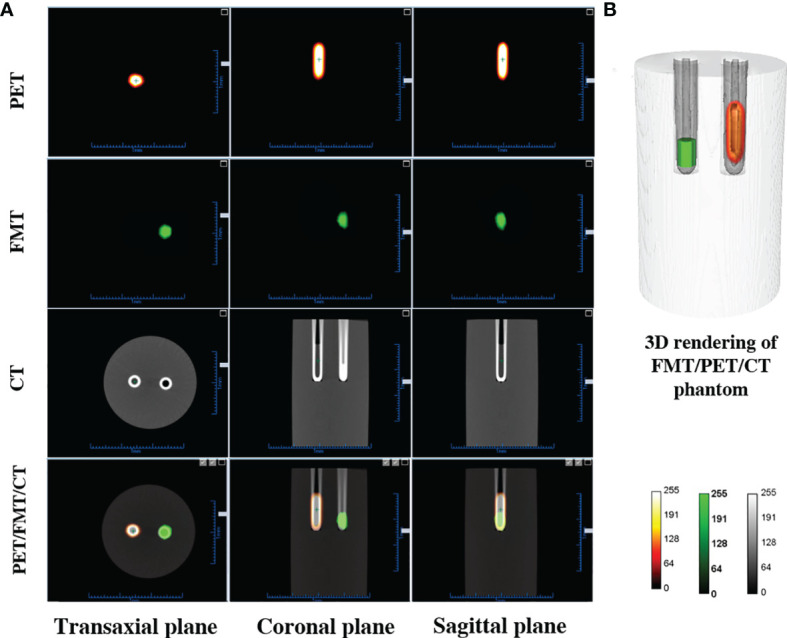
Results for the multi-modality imaging. **(A)** PET, FMT, CT, and tri-modality fusion images. **(B)** Three-dimensional rendering of fusion images demonstrating co-registration performance.

Reconstructed PET, FMT, CT, and tri-modality fusion images of the cylindrical phantom are shown in **Figure 3**. Three different color maps are used for the representation of the three different data sets in **Figure 3A**. The integrated tri-modality three-dimensional images are shown in **Figure 3B**. The ICG solution within the left capillary tube is displayed in green. The ^18^F-FDG within the right capillary tube is shown by a hot spot representation. Taken together, our results indicate that our multi-modality imaging system is able to provide fused images with a misalignment of less than 1 mm).

### *In Vivo* Imaging

**Figure 4:**
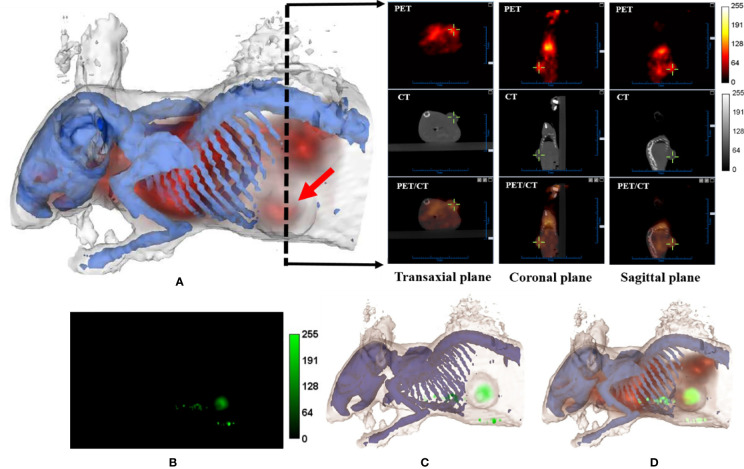
Multi-modality imaging analysis of nude mice bearing WSU-HN6 xenograft tumors. **(A)** Three-dimensional rendering of mouse bones and skin based on CT data using 3D-Slicer. The PET (apoptotic) signal is merged with the rendering volume (red arrow, tumor). The black dashed arrow indicates the position of transverse slices in CT, PET, and merged PET/CT images in the transaxial, coronal and sagittal plane. **(B)** Fluorescence image (ICG signal, corresponding to necrotic tumor area) of a mouse obtained by FRI. **(C)** Three-dimensional rendering of mouse bones and skin and fluorescence signal based on FRI. **(D)** Three-dimensional rendering of CT/PET/Fluorescence (red, apoptotic signal; green, necrotic signal).

After bacteria treatment, Balb/c nude mice bearing a subcutaneous WSU-HN6 xenograft were subjected to multi-modality imaging in order to examine the apoptotic and necrotic induction within the tumor, as well as to evaluate tumor growth compared to the control group *in vivo*. **Figure 4A** shows transverse, coronal, and sagittal slices of the small-animal PET/CT, as well as the X-ray image of the ear, bone, and tumor tissues in the lower, left rib, and several nodes of a single mouse.

To obtain better subcutaneous fluorescence data, the FRI mode was used in the animal study. Each rotational angle of the acquisition was recorded so that the FRI image could be successfully projected and merged with the CT images. The results indicated cell death in the center part of the xenograft WSU-HN6 (**Figures 4B, C**). The additional ICG signal, detected outside the tumor, could have been caused by the tumor or by bacteria obstructing a skin vessel.After the administration of the bacteria, apoptosis induction was examined in real-time by using an ^18^F-ML-10 tracer. The merged real-time images showing the apoptotic and necrotic tumor areas, including the whole-body animal structure, are shown in **Figure 4D**.

### A New Recombinant B.breve-IL-24 Strain Induces Tumor Apoptosis and Growth *In Vivo*

**Figure 5:**
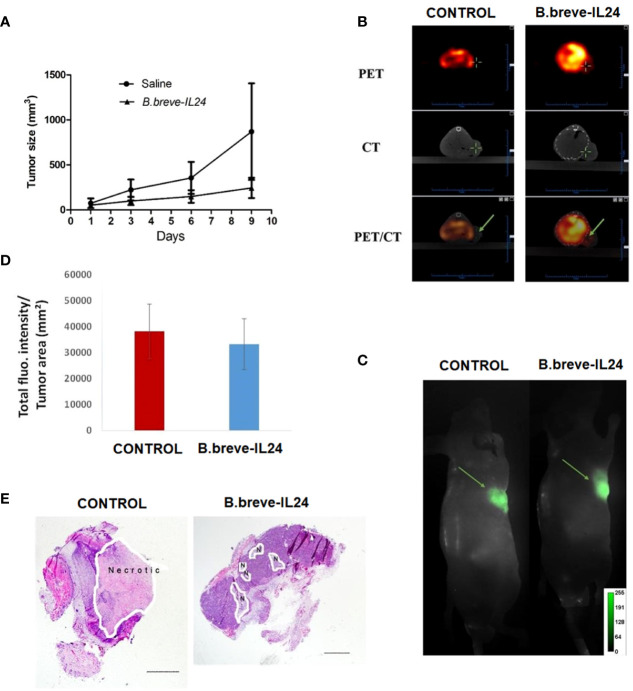
Growth curve, apoptosis induction, and necrotic tissue detection in mice bearing a WSU-HN6 tumor treated with **(B)** breve-IL24 *in vivo*. **(A)** Tumor growth curve over 2 weeks of therapy. **(B)** Tumor uptake of ^18^F-ML-10 tracer *in vivo* by PET/CT scanning; the detected radioactivity was 135.35 μCi for the treatment group (volume 169.13 mm³) and 20.09 for the control group (volume 129.96 mm³). **(C)** Real-time FRI images of the tumor necrotic area (ICG fluorescence) **(D)** quantification of total tumor fluorescence within the tumor in treatment and control group, p<0.045 **(E)** H&E staining of tumor tissues, scale bar=1mm.

To further evaluate the antitumor effect of B.breve-IL-24 *in vivo*, a xenograft mouse model was established. When tumors reached 8 to 10 mm in diameter, B.breve-IL-24 or saline (control) was injected. Results showed higher tumors grew in the control group compared to B.breve-IL-24-treated mice, which suggests that the B.breve-IL-24 strain can inhibit tumor growth over time (**Figure 5A**). Tumor size was measured to monitor the growth inhibition efficiency *in vivo*. Saline group was used as blank control and B. breve-GFP was used as negative control. A higher tumor growth inhibition was observed in B. breve-IL24 compared with B. breve-GFP group and higher tumor growth inhibition in B. breve-GFP compared with the Saline group. Although no significance between B. breve-GFP and B. breve-IL24 group were observed. Nevertheless, the average tumor size (including slower tumor growth rate) in B. breve-IL24 was smaller compared with the B. breve-GFP group.

Interleukin-24 (IL-24) is a novel tumor suppressor cytokine that selectively induces apoptosis in a wide variety of tumor types. To further substantiate an activation of the apoptosis pathway, we analyzed the effect of B.breve-IL-24 on caspase-3 activation in tumor tissues. We found that B.breve-IL-24 induced activation of caspase-3 and Bim in tumor tissues, the results could be found in our previous study (18).

The PET/CT scanning results of the tumors treated with B.breve-IL-24 showed a significantly higher apoptosis induction as detected by the higher radioactivity (135.35 µCi) compared to the control group (20.09 µCi) *in vivo* (**Figure 5B**). These results were confirmed by Western blotting of pro- and anti-apoptotic proteins. We could show that Bim and cleaved caspase-3 were highly expressed by B.breve-IL-24, while low Bim expression was detected in this strain, indicating a low Bcl-2 expression (18).

Finally, the necrotic tumor area was analyzed by bright field microscopy after histopathologic tissue staining. In contrast to the apoptosis induction results, a larger necrotic tumor area was observed in the control group (**Figures 5C–E**). The reason for the higher necrosis in the control group may be explained by the faster growth of the tumor compared to the treatment group.

In summary, our multi-modality system provides more comprehensive information about the tumor behavior and the therapeutic effect *in vivo* than single modality approaches.

## Discussion

Optical imaging is a technique that offers high sensitivity at low cost and short acquisition times. The combination of optical techniques with nuclear apoptosis imaging methods has been hampered due to the lack of a standard imaging system. Our custom-built integrated tri-modality imaging system combines two functional imaging modalities (PET and FMI) with an anatomic imaging modality (CT). In this study, we described the system’s technological development, the optimization strategy of the imaging protocols, and the system performance *in vitro* and *in vivo.* The fusion of different modality images provides the high anatomical resolution of a CT with a joint optical/PET molecular readout.

In the last decade, numerous potential probes, both proteins and small organic molecules, be they radioactive or non-radioactive (optical, magnetic resonance imaging and ultrasound), respectively, have been developed (24–28). However, it is difficult to predict which imaging modality fits best to any given apoptosis-directed chemical entity. For example, the use of near-infrared fluorescent reporters preserves their high affinity while high absorption and scattering in tissues would limit the applicability for tumor imaging purposes *in vivo*. On the one hand, PET provides robust in-depth detection capabilities, on the other hand, it requires the administration of a radioactive contrast agent. In any case, applications being able to detect tissues apoptosis would benefit from any imaging modality, no matter of which type. Therefore, current strategies in tissues apoptosis imaging rely on multi-modality approaches that combine the strengths and capabilities of different imaging techniques (2, 6). However, the fusion of nuclear and optical methods is presently an uncommon strategy because of some misconceptions and technical difficulties (29).

The three modalities (PET/CT/FMI) were integrated in the same animal bed and images were taken in a parallel scan leading to a more accurate registration compared to software-based methods used in single (separate) modality scans. Consequently, this integrated system offers a lower mouse throughput-to-cost ratio, a shorter scan duration and less motion artifacts. Therefore, the system provides synergistic advantages compared to single imaging modalities. In this study, a near infra-red fluorescent dye, ICG, was used in FRI, in order to provide information about tumor cell death (necrosis) while a novel PET tracer, ^18^F-ML-10, was used to further investigate apoptosis induction in malignant cells. In addition, the CT scan was used to examine the anatomical information of the animal body and bone structures. To conclude, our data suggest that the combination of these imaging tools might provide more accurate and comprehensive information about tumor behavior and the therapy success *in vivo*.

Another important part of this work is the induction of apoptosis in the pathogenesis and treatment of cancer disease. The electro-transformed bacteria Bifidobacterium breve was delivered into tumor tissues to induce apoptosis. Briefly, it has been shown that bacteria organism are able of replicating and targeting the hypoxic region of the tumor *in vivo*; usually only 3-5% of tumor cells are considered in the growth fraction while the rest 95% of tumor tissue is hypoxic to some degree (30, 31). The hypoxic regions of solid tumors provide some species of anaerobic bacteria such as Bifidobacterium and Lactobacillus a suitable environment to germinate and grow (32). So, the use of beneficial bacteria such as Bifidobacterium strains offers a straight-forward, safe, and clinically acceptable approach of delivering therapeutic proteins locally within the tumor environment, external to tumor cells. Over the last decade, a number of data has been published by using Bifidobacterium species such as Breve, Infantis, Longum and Adolescentis for liver, lung and melanoma tumor therapy *in vivo* (20, 21, 33). Apoptosis is an ordered and orchestrated cellular process that occurs in physiological and pathophysiological conditions. Cancer is one of the scenarios where too little apoptosis occurs, resulting in malignant cells that will not die (34).The mechanism of apoptosis is complex and involves many pathways (35). Despite being the cause of problem, apoptosis plays an important role in the treatment of cancer as it is a popular target of many treatment strategies (35, 36).The abundance of literature suggests that targeting apoptosis in cancer is feasible (37–41).Therefore, developing apoptosis imaging techniques *in vivo* are crucial for the evaluation of anti-tumor drug therapy efficacy (42). Although radionuclide-based molecular imaging of apoptosis is developing rapidly, there is no method to comprehensively characterize apoptosis because of the various underlying mechanisms. It is of great significance to develop new apoptosis imaging techniques in the future as well as translational research on the significance of imaging the different forms of cell death that are able to reduce non-specific background. In this article, we have investigated the use of health-promoting bacteria as a gene delivery system (IL-24 gene) for cancer therapy *in vivo*. MDA-7/IL-24 is a member of the IL-10 family and has been strongly correlated to apoptosis induction in a variety of cancers including melanoma, breast, liver, prostate, ovarian, and nasopharyngeal cancer (43–47). By using the tri-modality imaging system, we were able to simultaneously monitor the bacteria-induced therapeutic effect, comprising apoptosis and necrosis induction, on head and neck tumor xenografts *in vivo*. By using the tri-modality imaging system, we were able to simultaneously monitor the bacteria therapeutic effect, including the apoptotic and necrotic induction within the tumor, on head and neck xenograft *in vivo*. While, the apoptotic induction was examined in real-time by using 18F-ML-10 tracer, the cell death *in vivo* was detected using commonly known fluoresce probe ICG. In summary, our data demonstrate that radioactive and fluorescent signals from ^18^F-ML-10 and ICG could be clearly detected in whole-body animal experiments. Although the exact mechanism of plasma membrane depolarization has not yet been fully investigated, our results support the hypothesis of a selective binding of ^18^F-ML-10 to apoptotic cells in the treatment group but not in the control groups. These observations are consistent with previous preclinical and clinical studies (10, 48). Moreover, fluorescence reflectance imaging provided complementary information of necrotic tissues, tumor information that cannot be obtained by using micro-PET. Finally, with the help of a high spatial resolution CT and a high specificity of imaging probes, our system has been shown to be a powerful tool to monitor early tissues responses upon therapeutic intervention by being able to interpret tumor growth inhibition and tumor cell apoptosis induction. Both qualitative detection and quantitative determination in small animal molecular investigations is expected to provide much more information

## Conclusion

In this paper, a new integrated custom-built tri-modality system (PET/CT/Fluorescence) for small animals was developed. This system integrates structural, functional, and molecular imaging with a therapeutic approach. Furthermore, this new translational platform combines common optical techniques and nuclear medicine methods and represents the ultimate multi-modality platform in biomedical research, thus offering a powerful diagnostic approach to investigate the tumor behavior and therapy in real-time. Moreover, we have established and validated the use of a new recombinant bacteria B.breve-IL-24 gene for cancer therapy *in vivo*. The results from tri-modality system further confirm that B.breve-IL-24 could offer a novel, safe, and clinically acceptable therapeutic approach for tumor therapy *in vivo*, which might contribute to preoperative prediction and intraoperative monitoring of tumors.

## Data Availability Statement

The original contributions presented in the study are included in the article/**Supplementary Material**. Further inquiries can be directed to the corresponding authors.

## Ethics Statement

The animal study was reviewed and approved by Peking University ethic committee.

## Author Contributions

YK, XZ and SL designed the idea and finished the manuscript. ZX contributed to designing the three-modal image system. IV, LW and KZ performed the biochemistry and immunology experiments. ZP helped to elaborate the content about cell apopsis and checked the expression. XZ and SL analyzed the data. YK, ZX and QR supervised. YK, XZ, SL, IV and LW revised the manuscript based on reviewers' comments. All the authors had read the manuscript and helped to improve the expression. All authors contributed to the article and approved the submitted version.

## Funding

This work was supported by The National Key Instrumentation Development Project (2011YQ030114), the National Basic Research Program of China (973 Program, 2011CB707500), and the National Natural Science Foundation of China (11104058).

## Conflict of Interest

The authors declare that the research was conducted in the absence of any commercial or financial relationships that could be construed as a potential conflict of interest.

## Publisher’s Note

All claims expressed in this article are solely those of the authors and do not necessarily represent those of their affiliated organizations, or those of the publisher, the editors and the reviewers. Any product that may be evaluated in this article, or claim that may be made by its manufacturer, is not guaranteed or endorsed by the publisher.
