# Supplementary material for: A Hybrid Imaging Platform(CT/PET/FMI) for Evaluating Tumor Necrosis and Apoptosis in Real-Time
Source: Front Oncol. 2022 Jun 22;12:772392. doi: 10.3389/fonc.2022.772392 (PMC9257022; doi:10.3389/fonc.2022.772392)
Supplement: Supplementary file 1 [file DataSheet_1.docx]

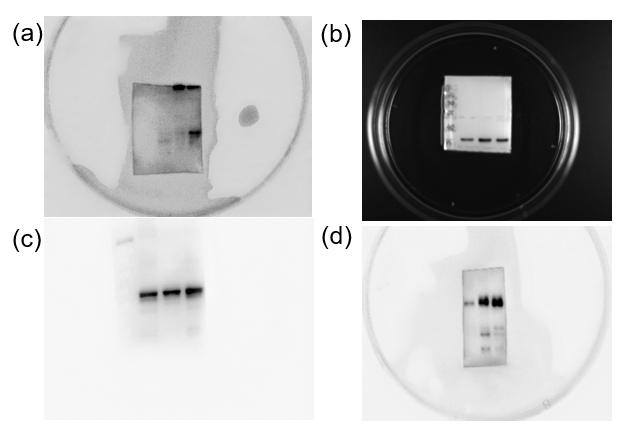


**Supplementary FigureS1. The original figure of Western Blot. The antibodies used for each group (a)cleaved caspace 3 (b) GADPH (c) t-caspace3 (d)bim. All the blots were run at the same SDS-PAGE and membrane transfer.**


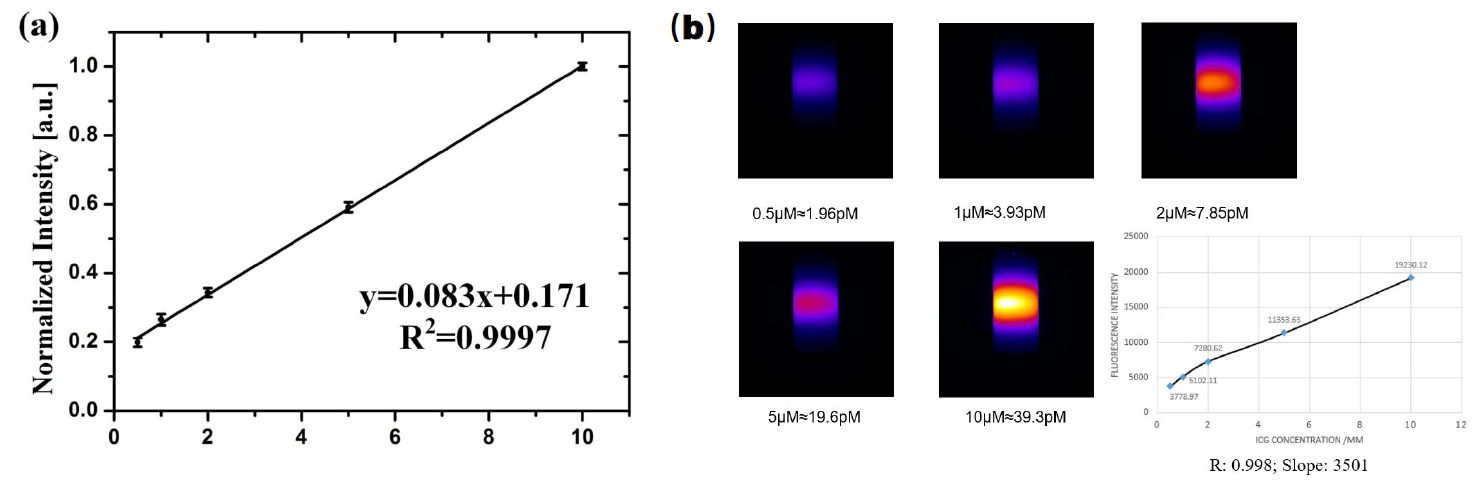


**Supplementary FigureS2. The linear range of the fluorophore test. (a) Tests of linearity with ICG in 1% intralipid solution; (b) the association between ICG brightness and ICG concentration**


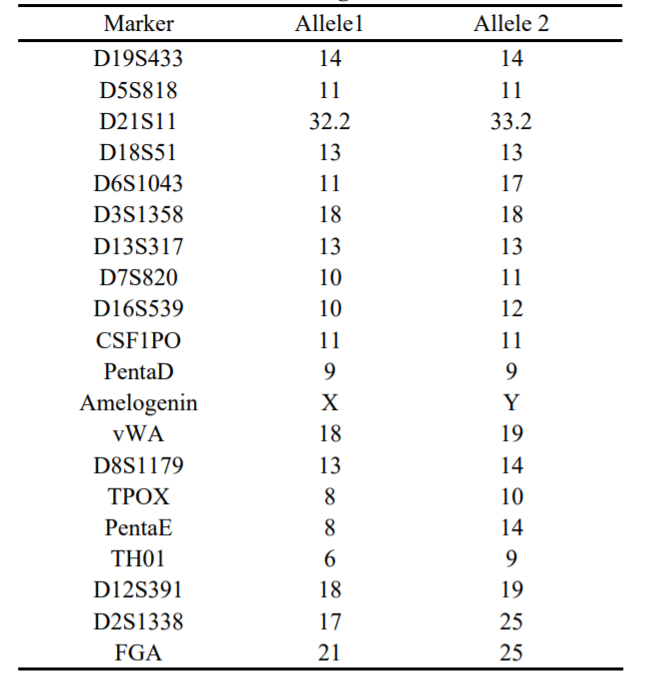


**Supplementary FigureS3. The STR profiling of WSU-HN6 cell line. This information was first published in the supplementary Fig 4 of “Bifidobacterium breve as a delivery vector of IL-24 gene therapy for head and neck squamous cell carcinoma in vivo” by Wang et.al. in Gene Therapy (2017).**

**Supplementary Note**

Method: Western blot test

Tumor tissues were carefully dissected and stored in liquid nitrogen, followed by lysing in RIPA buffer (Applygen, Beijing, China) containing proteinase inhibitors and phosphatase inhibitors. After measuring protein concentration using the BCA kit (Thermo Fisher Scientific, Waltham, MA USA), equal amounts of protein samples were separated by 15 % sodium dodecyl sulfate–polyacrylamide gel electrophoresis (SDS-PAGE) and transferred to polyvinylidene difluoride membranes by wet blotting. The membranes were blocked in 5 % non-fat dry milk for 1 h and probed with antibodies against cleaved caspase-3 (CST, 1/1000), Bim (Gentex, 1/1000), Bcl-2 (CST,1/1000) and GAPDH (CST,1/1000) separately at 4 °C overnight. After incubation with peroxidase-linked secondary antibodies for 1 h at room temperature, the enhanced chemiluminescent (ECL) reagent was used to visualize the immune-reactive proteins.

Histopathology assay

[Hematoxylin and Eosin Staining](http://www.biotek.com/resources/articles/hematoxylin-and-eosin-stained-tissue.html) used 3μm-thick sections cut from formalin-fixed paraffin-embedded tumor tissue samples and followed standard procedures. Briefly, after deparaffinize and re-hydrate in gradient alcohol, slides were stained in Herris hematoxylin for 1min and then ran in tap water. Continued by counterstain in eosin-phloxine solution for 30s and dy-hydrate through 95% alcohol and absolute alsocol and clear in xylene and mounted.
